# Numerical Model of Streaming DEP for Stem Cell Sorting

**DOI:** 10.3390/mi7120217

**Published:** 2016-11-30

**Authors:** Rucha Natu, Rodrigo Martinez-Duarte

**Affiliations:** Multiscale Manufacturing Laboratory, Department of Mechanical Engineering, Clemson University, Clemson, SC 29634, USA; rnatu@g.clemson.edu

**Keywords:** streaming dielectrophoresis, neural stem cells, numerical simulation

## Abstract

Neural stem cells are of special interest due to their potential in neurogenesis to treat spinal cord injuries and other nervous disorders. Flow cytometry, a common technique used for cell sorting, is limited due to the lack of antigens and labels that are specific enough to stem cells of interest. Dielectrophoresis (DEP) is a label-free separation technique that has been recently demonstrated for the enrichment of neural stem/progenitor cells. Here we use numerical simulation to investigate the use of streaming DEP for the continuous sorting of neural stem/progenitor cells. Streaming DEP refers to the focusing of cells into streams by equilibrating the dielectrophoresis and drag forces acting on them. The width of the stream should be maximized to increase throughput while the separation between streams must be widened to increase efficiency during retrieval. The aim is to understand how device geometry and experimental variables affect the throughput and efficiency of continuous sorting of SC27 stem cells, a neurogenic progenitor, from SC23 cells, an astrogenic progenitor. We define efficiency as the ratio between the number of SC27 cells over total number of cells retrieved in the streams, and throughput as the number of SC27 cells retrieved in the streams compared to their total number introduced to the device. The use of cylindrical electrodes as tall as the channel yields streams featuring >98% of SC27 cells and width up to 80 µm when using a flow rate of 10 µL/min and sample cell concentration up to 10^5^ cells/mL.

## 1. Introduction

Next-generation therapeutics such as cellular therapy and tissue regeneration are largely based on the use of stem cells [1]. These cells are characterized by a varying capacity for growth and the ability to either differentiate into specialized cells or maintain their stem cell phenotype. Neural stem cells are of special interest given their potential for neurogenesis to treat spinal cord injuries and other nervous disorders [2,3,4]. Neural stem cells can be differentiated as three types of cells in the central nervous system: neurons, astrocytes, and oligodendrocytes [5], with neurons being the most sought-after type of cell. Neurons obtained from the neurogenic progenitors play an important role in the treatment on Parkinson’s disease, spinal cord injuries, and motor neuron diseases and also to restore lost neuronal populations [6]. However, the dynamic nature of stem cells and their susceptibility to environmental changes demand technology to monitor, characterize, and manipulate living cells [7].

Currently, the most common methods used to quantitatively characterize stem cells include fluorescence-activated cell sorting (FACS) [8], magnetic bead-coupled cell separation [9] and micropipette aspiration [10]. These methods normally use specific labels or formulation of certain probes for detection of the stem or differentiated cells. However, there is no clear set of surface markers with sufficient specificity to identify promising cells, such as neural stem cells, from a background. This limits investigation of lineage-biased progenitors and their potential use as therapeutic agents. Hence, alternatives to traditional techniques are needed. The use of membrane capacitance and dielectrophoresis (DEP) as a label-free technique to discriminate targeted cells from a background is presented here. We focus on studying the impact of device design and experimental protocol on the throughput and efficiency when separating neurogenic progenitor cells (SC27) from astrogenic ones (SC23) in a sample of neural stem/progenitor stem cells (NSPCs). The study presented here is based on the experimental results by Labeed and co-workers where they demonstrated that the capacitance of neurogenic progenitors, SC27, differs from astrogenic progenitors, SC23, in a human NSPC population even when their size is virtually identical. Furthermore, they demonstrated that membrane capacitance increases with age of NSPCs [11]. The ultimate goal is an in-line module for cell sorting that could be incorporated in the bio manufacturing of therapeutic cells, such as after cell expansion [12].

Dielectrophoresis (DEP) refers to movement induced on the cells by an electric field gradient [13]. When the cell is less polarized than the medium, the cell moves away from the field gradient and such movement is commonly known as negative DEP. When the cell is more polarized than the medium, it moves towards the field gradient, which is known as positive DEP. Hence, by tailoring such behavior, one can direct the cells to specific locations to allow for sorting. For example, cells can be attracted to the electrodes using positive DEP. The response of the cell to a polarizing field gradient of a given frequency and when suspended in a specific medium depends on its membrane capacitance as detailed in the theoretical section below for frequencies below the MHz range. The membrane capacitance of a cell depends on its surface area and surface topography, which change as a response to various internal processes and external stimuli. Thus, membrane capacitance is used as a label-free, non-destructive quantitative indicator of cell identity and in combination with DEP to enable cell separation [11,14,15,16,17,18,19]. For example, besides their work with human stem cells, Labeed and colleagues have demonstrated the separation of mice neurogenic progenitors from a mixture [11] while Bagnaninchi et al. used membrane capacitance to detect differentiated adipocytes and osteoblasts from their progenitors [19]. Membrane capacitance has also been used to identify six main leucocyte subpopulations in hematopoietic lineage [18], while Stephens et al. have characterized how the membrane capacitance of *Clostridium difficile* can be used for separation from peripheral blood cell harvest and Talary et al. demonstrated the separation and enrichment of hematopoietic stem cells that express CD34+ from bone marrow and peripheral blood [20,21]. Furthermore, Flanagan et al. concluded that distinct changes in the dielectrophoretic properties of the neural stem cells are observed before the presence of specific cell-surface proteins (antigens) can be detected [7] and Vykoukal et al. used DEP coupled with field flow fractionation to enrich putative stem cells from adipose tissue [22].

In this paper, we use numerical simulation to assess the potential for continuous separation of Human Neural Stem/Progenitor Cells (HuNPSC) using streaming DEP. Streaming DEP refers to the focusing of targeted cells into specific streams to facilitate their retrieval from the channel. The width of the stream should be maximized to increase throughput while the separation between streams must be widened to increase efficiency during retrieval. The goal is to understand the factors that impact the forming of these streams. Here we are interested in forming streams of neurogenic progenitors (SC27) to facilitate their separation from astrogenic (SC23) progenitor cells. To this end, we assess the impact of electrode geometry, experimental flow rate and sample concentration on the efficiency and throughput of streaming DEP. We define efficiency as the ratio between the number of SC27 cells over total number of cells retrieved in the streams, and throughput as the number of SC27 cells retrieved in the streams compared to their total number input to the device. The implemented model can be applied for different electrode materials and DEP technologies. However, our goal is to use this model for the optimization of carbon electrodes. These glass-like carbon electrodes are made by carbonization of SU-8 photoresist and have been demonstrated in a number of DEP applications [23,24,25,26,27].

## 2. Operating Principle of Streaming Dielectrophoresis (DEP)

**Figure 1:**
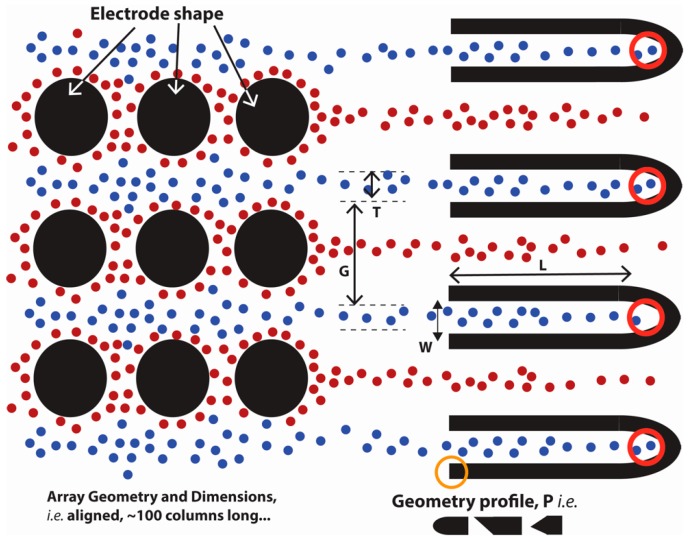
Schematic of the working principle of streaming dielectrophoresis (DEP). Cells can be focused into streams of specific width *T* and separation *G* between each other. Retrieval geometries can be used for continuous extraction from the channel. The impact of device geometry and experimental parameters on the composition and width of the streams is studied here. Specifically, we study the impact of electrode shape (circles, diamonds, lens-shaped) and height (10%, 50% and 100% of the channel height) as well as flow rate and cell concentration in the sample.

Cells can be attracted to a field gradient around the electrodes using positive DEP or repelled from it using negative DEP. The theory behind this is shown in the next section. Streaming DEP is the focusing of targeted cells into streams either collinearly with the electrodes by using positive DEP or between the electrodes using negative DEP. This is illustrated in Figure 1, where the red particles are attracted to the electrodes and eluted away in lines collinear with the electrodes. This is due to the fact that the drag and DEP forces acting on the cell are in equilibrium. Increasing the DEP force will lead to particle trapping while increasing the drag force. Increasing flow velocity will lead to hydrodynamic focusing only. The combination of DEP with hydrodynamic focusing is desired to increase the resolution of the system since hydrodynamic focusing depends mainly on the particle size [28]. The blue particles in the figure are focused in streams between electrodes using negative DEP, as in this case is where the field gradient is of less magnitude. Once focused in streams, the retrieval of cells can be facilitated by geometries as those shown in the figure. By choosing the frequency of the polarizing waveform appropriately, one can induce a positive DEP force on specific cells while inducing negative DEP on the rest. Here, we will study how the device geometry, such as electrode cross-section and height relative to the channel, and experimental parameters, such as flow rate and sample concentration, impact the width *T* and cell composition of these streams. We strive to understand how these parameters need to be optimized to enable streams featuring all of the SC27 cells introduced in the system (100% throughput) without contamination with SC23 cells (100% efficiency). The design of the retrieval geometries is beyond the scope of the work presented here.

## 3. Materials and Methods

### 3.1. Theoretical Framework

**Figure 2:**
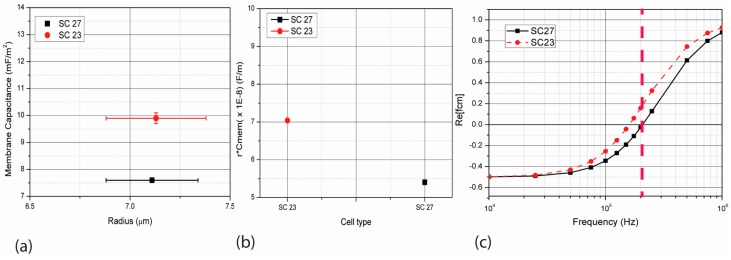
(**a**) The radius and membrane capacitance of different cells considered in this work as obtained experimentally by Labeed et al. [11]. The membrane capacitance of SC23 cells is 9.9 ± 0.2 mF/m^2^ while that of SC27 is 7.6 ± 0.3 mF/m^2^. The cell radius lies between 6.57 and 7.75 µm; (**b**) The product of radius and membrane capacitance for SC23 and SC27 is different and can be used to further distinguish the two types of cells. The values of *rC*_mem_ for SC27 and SC23 used here are (5.40 ± 0.06) × 10^−8^ F/m and (7.50 ± 0.05) × 10^−8^ F/m respectively. Note the small deviation on the average values (**c**). The values of the real part of the Clausius Mossotti factor *Re*[*f*_CM_] for SC23 and SC27 as frequency changes are obtained using Equation (2) using the *r* and *C*_mem_ values shown in Figure 2a. At the frequency of 200 kHz (dashed red line), the cells SC23 and SC27 show the highest difference in their *Re*[*f*_CM_] values, whereas SC23s show a positive value of 0.15, and SC27s show a slight negative −0.02.

Labeed et al. [11] have shown how the membrane capacitances of SC27 and SC23 huNSPCs are different even though they are remarkably similar in their size and levels of nestin and SOX2 markers. Their experimental results are replicated here in Figure 2 with SC23 cells displaying a membrane capacitance value of 9.9 ± 0.2 mF/m^2^ and SC27 cells of 7.6 ± 0.3 mF/m^2^ and both overlapping in the size range of 6.57 to 7.75 µm.

Dielectrophoresis is the motion of electrically polarizable particles induced by the interaction between an electrical dipole induced on the cell and an electric field gradient [13]. The product *rC*_mem_ gives the cells a specific signature which will dictate the movement of the cell either towards, positive DEP, or away, negative DEP, from the electric field gradient established around the electrodes. The DEP force *F*_DEP_ for a spherical particle is given by Equation (1) and depends on the size of the particle *r*, gradient of the square of the electric field $\nabla E_{\mathrm{rms}}^{2}$, permittivity of the medium ε_m_ and the magnitude of the real part of the Clausius Mossotti factor *f*_CM_.

(1) $$F_{\mathrm{DEP}}=2\pi r^{3}Re\left[f_{\mathrm{CM}}\right]\nabla E_{\mathrm{rms}}^{2}$$

Several authors have detailed the use of the crossover frequency method to determine the Clausius Mossotti factor of a cell experimentally [29,30]. In this method, *Re*[*f*_CM_] is given by Equation (2):

(2) $$Re\left[f_{\mathrm{CM}}\right]=\frac{{\left(\sqrt{2}\pi rC_{\mathrm{mem}}f\right)}^{2}-\sigma_{m}^{2}}{{\left(\sqrt{2}\pi rC_{\mathrm{mem}}f\right)}^{2}+2\sigma_{m}^{2}}$$

where *f* is the frequency of the polarizing signal and σ_m_ is the electrical conductivity of the suspending medium. The dependence of *Re*[*f*_CM_] on frequency for SC27 and SC23 cells when suspended in a medium with conductivity of 0.05 S/m is calculated using Equation (2) and shown in Figure 2c when using values of *r* and *C*_mem_ obtained experimentally by Labeed et al. [11]. A polarizing frequency of 200 kHz is used in this work to model separation between different cell types, since at this frequency the difference between the *Re*[*f*_CM_] of SC23 (0.15) and SC27 (−0.02) is the maximum.

In order to implement continuous separation using cell focusing into elution streams by streaming DEP, the interaction between DEP force and hydrodynamic drag acting on the particles must be analyzed. The Stokes drag force *F*_DRAG_ is given by Equation (3) where the dynamic viscosity of the medium is η, the radius of the spherical particle *r*, the flow velocity *u* and the particle velocity *v*.

(3) $$F_{\mathrm{DRAG}}=6\pi \eta \left(u-v\right)$$

The sedimentation force on the cells resulting from gravity and cell buoyancy is also important and is given by Equation (4), where $F_{s}$ is the sedimentation force, $d_{p}$ is the density of the cell, *d* is the density of the medium and *g* is the gravitational constant.

(4) $$F_{s}=\frac{4}{3}\pi r^{3}\left(d_{p}-d\right)g$$

Hence, the motion of the particle in the microfluidic channel is influenced by the total force $F_{t}$ which is calculated by vector summation of *F*_DEP_, *F*_DRAG_ and *F*_s_:

(5) $$F_{t}=F_{\mathrm{DRAG}}+F_{\mathrm{DEP}}+F_{s}$$

According to Newton’s second law of motion, the total force $F_{t}$ can also be expressed as,

(6) $$m_{p}\frac{dv}{dt}=F_{t}$$

where the mass of the particle is *m*_p_ and *v* is particle velocity. The total force acting on a particle is given by Equation (7) by combining the Equations (1)–(6)

(7) $$m_{p}\frac{dv}{dt}=6\pi \eta r\left(u-v\right)+2{\pi \varepsilon }_{m}r^{3}Re\left[f_{\mathrm{CM}}\right]\nabla E_{\mathrm{rms}}^{2}+\frac{4}{3}\pi r^{3}\left(d_{p}-d\right)g$$

Equation (7) can be considered a general equation from which the sum of forces on a particle can be obtained. After proper manipulation [17], the vector of particle velocity can be obtained with Equation (8)

(8) $$v=u+\frac{F_{DEP}+F_{s}}{6\pi \eta r}$$

where ***v*** is the particle velocity vector at a given point, ***u*** is the flow velocity vector; ***F*_DEP_** is the force vector given by Equation (1) for a specific cell and experimental conditions; and *F*_s_ is the sedimentation force assuming a constant density throughout the medium. The 3D flow velocity field in the device as well as the electric field gradient that is required to calculate the DEP force acting on a particle throughout the device are calculated using COMSOL Multiphysics (Version 4.4, Stockholm, Sweden). The particle velocity vectors are then used to compute the position of the particle by the Lagrangian tracking method and originate streamlines of potential particle trajectories in the device [31].

There are two other important considerations that need to be taken into account when designing devices for cell sorting. The first one is the effect of shear stress on the cell. Shear stress is the tractive force produced by a moving viscous fluid on a solid body in relative motion with the fluid [32]. Although physiological levels of shear stress in the aortas of adults and embryos have been observed as 15 and 5 dyne/cm^2^ respectively, magnitudes down to 1.5 dyne/cm^2^ are known to effect adult blood phenotypes [33,34,35]. Exposure of embryonic stem cells to shear stress values of 1.5 to 15 dyne/cm^2^ for 2 days promoted differentiation of embryonic stem cells [36]. Since the intended application of this work is the sorting of neurogenic progenitors, a conservative upper limit of shear stress on the cell is taken as 1.5 dyne/cm^2^. This reduces the possibility of introducing undesired changes in the manipulated cells. It is important to note that the transit time of cells through the device when using streaming DEP will likely be in the order of few seconds and thus long exposure to shear stress is not taken into account here.

The other important consideration is the impact of the electric field on the cell. For example, Salmanzadeh et al. reported on DEP experiments using insulator structures (insulator-based DEP or iDEP) where tumor cells remain viable after a 30 min exposure to an electric field gradient squared around 10^14^ V^2^/m^3^ in the frequency range of 100–600 kHz [37]. Su et al. conducted DEP trapping of the anaerobic microbe *Clostridium difficile* on insulator-based electrodes using similar gradient conditions, thoughin the frequency range of 0.1–5 MHz. Cell viability was intact after repeated occasional exposure to the field swept over 5 min for three hours [38]. In the case of manufacturing therapeutic cells, maintaining cell viability is obviously required but not enough since changes induced on the manipulated cells can later impact the properties of their progeny. Lu et al. reported that neural stem/progenitor cells suspended in a medium with conductivity of 0.01 S/m undergo undesirable changes in their DNA when exposed to a gradient of electric field squared at around 10^14^ V^2^/m^3^ at frequencies in the range near the crossover frequency of the neural stem/progenitor which is 50–100 kHz [39]. In this work, the range of the crossover frequency of cells is 100–200 kHz and a similar behavior is expected. Hence, a minute is taken here as the upper limit in the exposure time of the cells to the field gradient.

### 3.2. Computational Model

**Figure 3:**
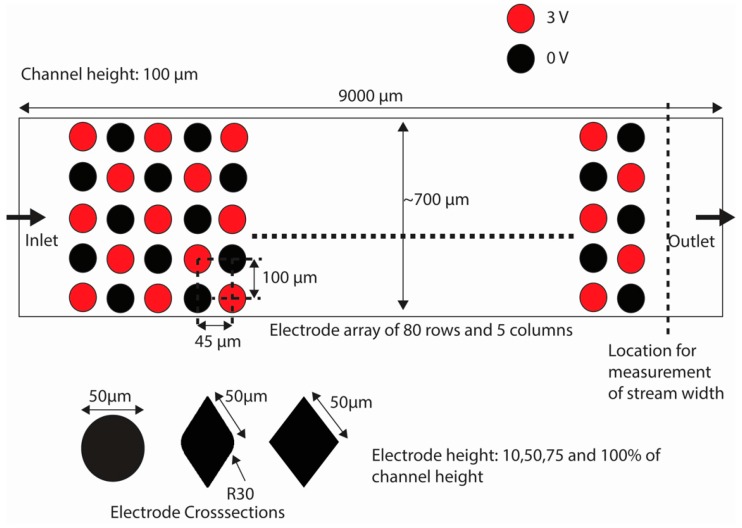
The top view of the model used for numerical analysis with the dimensions and electrode polarity is shown in the figure. The electrodes are arranged in a manner that they lie in straight rows and symmetrical rows. However, the electrodes are polarized alternately using either 3 or 0 V. The three cross-sections studied in this work are shown: circles, lens and diamonds. The model shown is not true to the scale, but represents the actual shape of the electrodes and channel.

COMSOL Multiphysics 4.4 (Stockholm, Sweden) was used to build the model to solve for the flow field and electric field in a microfluidics channel containing an array of electrodes. An Intel^®^ Xeon^®^ CPU E5-1650 v2 @ 3.50 GHz processor with a RAM of 32 GB and a 64-bit operating system was used for these simulations. The simplified top view of the geometrical model used is illustrated in Figure 3. The dimensions of the model are based on experimental DEP devices made with carbon electrodes and validated using different cells [24,40,41,42]. 3D electrodes spanning 10%, 50%, 75% and 100% of a 100 µm-high channel were simulated. The length of the channel was 9000 µm. Three types of electrode cross-sections were used: circle, diamond and lens. The characteristic dimension for each of them was 50 µm as diameter for circles and side for lens and diamond (see Figure 3). The channel width was around 700 µm and varied slightly depending on the shape of the electrode since the distance between the outer edge of the array and the channel wall was kept constant at 45 µm. The surface of the electrodes was arbitrarily polarized with voltages of 0 and 3 V to maintain a maximum electric field of 9.69 × 10^4^ V/m and field gradient of 8.49 × 10^14^ V^2^/m^3^ to reduce the possibility of changes to the cells [43]. Flow rates from 5 to 20 µL/min were implemented since a flow rate of 20 μL/min was calculated to be the maximum value before inducing shear stress above 1.5 dyne/cm^2^ on cells using the channel and electrode dimensions explored here. Given the length of the channel and flow rates simulated here, the maximum residence time of the cell in the channel is 40 s when using a flow rate of 1 µL/min.

The Laminar Flow and Electric Currents physics modules available in the software were used to compute the flow velocity and electric field gradient to calculate the drag and DEP force fields acting in the simulation domain at steady state. Gravitational and buoyancy forces were used to consider cell sedimentation. Equations presented in the theory section were solved to obtain the force fields. A mesh featuring ~4.0 million tetrahedral elements was implemented and controlled by the fluid flow physics. The average element quality achieved was 0.66. The grid used is a fine-meshed COMSOL grid with the maximum element size of 20 µm and minimum element size of 1.5 µm. Simulations were also performed with the maximum element thicknesses of 15 and 7.5 µm. The stream widths were calculated in each of these cases and the maximum deviation in stream width was calculated to be <6 µm. Since this error is smaller than the radius of the cells of interest here, the stream width does not vary significantly to affect the cell retrieval. Grid value is restricted by limited memory sources available for this study. Given the limited computational power available to us and the desire to model high cell concentrations, we did not model cells as spherical particles moving through the device. Instead, we modeled potential cell trajectories as the streamlines obtained by taking the particle velocity vectors calculated using Equation (8) and used them to compute the position of the particle by the Lagrangian tracking method [31]. Both flow velocity and electric field gradient were obtained using COMSOL Muliphysics. The starting points of as many streamlines necessary to simulate a given cell concentration in the channel were randomly determined by generating random values in MATLAB (2014a, MathWorks, Natick, NA, USA) and feeding them back to COMSOL as starting positions. Such protocol enabled the simulation of cell dispersion throughout the channel cross-section at the inlet as will be expected in experiments. At least three different simulations were performed for each case of interest. Random starting points were used for each of these cases and hence the error bars shown in the results. From this point on in this manuscript, we refer to cell trajectories and locations as those that can be expected from modeling the streamlines in the channel.

The fact that the cells are simulated as streams introduces certain limitations in the calculations of stream widths. The thickness of the cell is not taken into account while simulating the streams. Hence, two streams which can come very close to each other (closer than the value of cell diameter) will not appropriately depict cell behavior. Also, the number of cells that can be trapped on the electrodes is restricted. This is because as the cells accumulate at the electrode, they cover a certain positive area of the electrode. As more and more cells attract towards the electrode, less area is available for cell trapping. This restriction is not present in the case of the streamlines. In spite of these limitations, the streamlines help to depict potential paths taken by individual cells in the channel.

All the streams were released in the channel at one time with the inlet velocities depending on their starting position and corresponding to a parabolic flow profile in the channel. Furthermore, the electric field was assumed to not be affected by local field gradients around electrodes derived from particles already trapped on the electrode surface. All modeled materials were considered to be non-porous, while the properties of the medium are uniform throughout the channel. Since flow occurred at Reynolds numbers lower than unity, a creeping flow was assumed. The electrodes and channel boundaries were assigned a slip condition. This consideration was to address the experimentally observed behavior of the cells slipping around the electrode and channel surfaces rather than abruptly sticking to them when coming in contact with such a boundary. We initially implemented a no-slip boundary condition, but this led to streamlines abruptly terminating on electrodes and channel walls, which is not true to experimental observations when using different cell types [24,40,44]. Cells are only observed to be held on the surface electrode against a flow when the DEP force is significantly higher than the drag force. However, the cells are released and eluted as soon as the electrodes are de-polarized and the DEP force disappears.

The values for *Re*[*f*_CM_] of SC23 (0.15) and SC27 (−0.09) corresponding to the frequency of 200 kHz were used. This value was chosen based on Figure 2c which shows that the *Re*[*f*_CM_] values for the two types of cells show maximum difference; hence DEP force acting on the two types of cells will differ significantly. The values of radius for the different cells were also taken as shown in Figure 2a. Other simulation parameters are specified in Table S1 in Supplementary Information.

The electric field in the domain was derived using the equations solved in the electric currents domains, for a stationary study as,

(9) E=−∇V

where *E* is the electric field computed in the domain and *V* is the voltage assigned to the electrode surface. X, Y and Z components for *F*_DEP_ were calculated here by using the equations in Table S2 (in Supplementary Information) for Electric Currents in stationary domain for the SC23 and SC27 cells.

The flow velocity *u* for the laminar flow field was calculated using Equations (10) and (11) where fluid density is denoted by ρ, *u* is the flow velocity, *I* is the identity matrix, and *T* represents the transpose of the matrix.

(10) $$\rho \left(u\cdot \nabla \right)u=\nabla \cdot \left[-\rho I+\mu (\nabla u)+{\left(\nabla u\right)}^{T}\right]+F_{t}$$

(11) ρ∇⋅u=0

These values for the flow velocity and the *F*_DEP_ were used to simulate the particle motion with the particle velocity *v* given by Equation (8). Streamlines were simulated by using the velocity represented by Equation (8) with different inlet positions of the cells. The medium conductivity used was 0.05 S/m which has been shown to not affect the cell viability of stem cells, as was recently shown by Lu et al. as long as the cell is exposed for less than a minute [39].

### 3.3. Data Analysis

COMSOL data points corresponding to the ends of the streamlines were exported to MATLAB 2014a for post processing. MATLAB was used to filter the streams which represent the SC23 and SC27 cells that get captured at the electrodes and only the streams which reach the outlet were selected. Plots were generated for the end points of the streamlines at the channel outlet. This data is converted to a histogram wherein the channel width is divided into small bars (or datasets) for the range of around 6 µm width along the channel. This width was arbitrarily selected. Thus, the channel of ~700 µm-width is divided into around 115 bars. The number of streamlines reaching the outlet in the width of such a bar is stored and this data is used for calculating the percent purity of both SC23 and SC27 types of streams in the domain. This is calculated using Equation (12), where *X* can take the value of 3 and 7 depending on the cell type being analyzed (SC23 or SC27). Equation (13) is used to calculate the throughput of the system.

(12) $$\%\;\mathrm{purity}\;\mathrm{of}\;\mathrm{SC}2X\;\mathrm{cells}\;\mathrm{in}\;a\;\mathrm{bar}:\;\frac{\mathrm{total}\;\mathrm{number}\;\mathrm{of}\;\mathrm{SC}2X\;\mathrm{streamlines}\;\mathrm{in}\;\mathrm{the}\;\mathrm{bar}\times 100}{\mathrm{total}\;\mathrm{number}\;\mathrm{of}\;\mathrm{streamlines}\;\mathrm{in}\;\mathrm{the}\;\mathrm{bar}}$$

(13) $$\%\;\mathrm{throughput}\;\mathrm{of}\;\mathrm{SC}2X\;\mathrm{cells}=\frac{\mathrm{total}\;\mathrm{number}\;\mathrm{of}\;\mathrm{SC}2X\;\mathrm{streamlines}\;\mathrm{in}\;\mathrm{the}\;\mathrm{bar}\times 100\%}{\mathrm{total}\;\mathrm{number}\;\mathrm{of}\;\mathrm{streamlines}\;\mathrm{of}\;\mathrm{SC}2X\;\mathrm{at}\;\mathrm{inlet}}$$

## 4. Results

**Figure 4:**
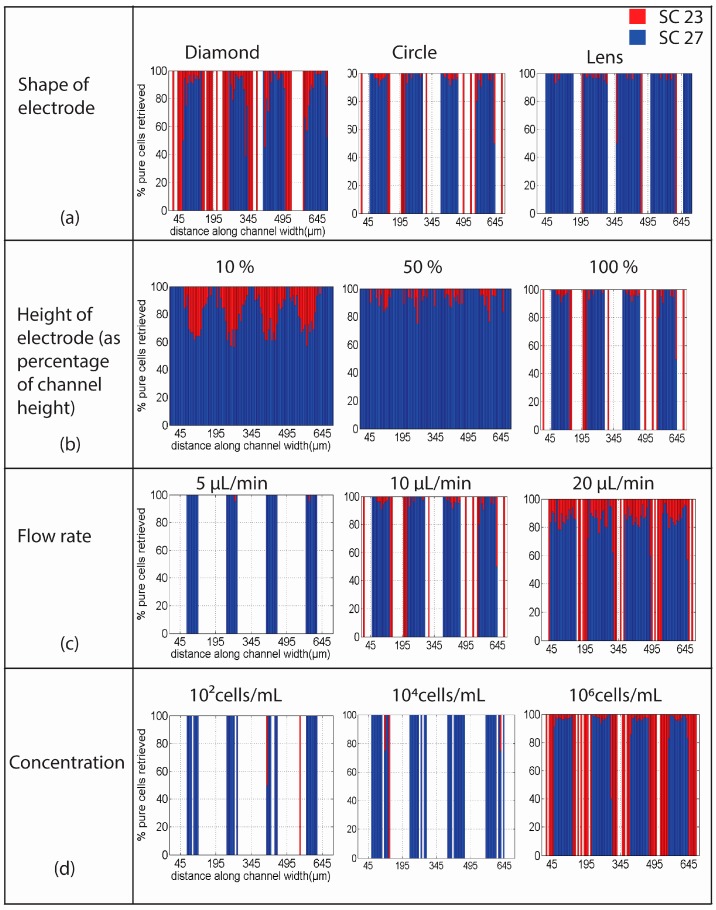
The percentage of SC23 and SC27 retrieved in different conditions considered in the numerical simulations. The stacked histogram in *y*-axis shows the percentage of SC23 and SC27 streams retrieved at different channel locations (from 0 to 700 μm). The *x*-axis in each case denotes the distance along the channel width and the grid points at 45, 195, 345, 495 and 645 μm mark the position of the center of each electrode in the channel. (**a**) The impact of electrode cross-section on cell separation; (**b**) Impact of electrode height relative to the channel. The purity of the streams increases with the increase in electrode height; (**c**) Impact of flow rate. 100% pure streams of SC23 cells can be obtained linearly to the electrodes as the flow rate increases. The purity of SC27 decreases at the same time; (**d**) Impact of cell concentration on the separation efficiency between cell types at 10 μL/min. SC23 cells are trapped at low concentrations and are not received, the array becomes saturated at high cell concentrations and SC23 streams start appearing collinear with the electrodes.

The results are resumed in the graphs shown in Figure 4a–d for different cases of electrode shape or cross-section, electrode height, flow rate and cell concentration in the sample. The *x*-axis in each case shows the distance along the channel with the scale marks denoting the center position of the electrodes (45, 185, 345, 495 and 645 µm). The percentage of SC23 (red) and SC27 (blue) streamlines flowing at that particular location is denoted in the *y*-axis. The cells of interest here are SC27 cells, given their neurogenic properties. A blue stripe spanning the complete height of the graph marks the specific location where a 100% pure SC27 cell stream can be retrieved. We calculated throughput as the ratio between the number of SC27 in the streams and those entered into the system. Hence, a 100% throughput means recovery of all cells entered in the system. The throughput is reported in Table 1. The goal of this work is to study the impact of different electrode geometries and experimental parameters on the efficiency and throughput of streaming DEP.

### 4.1. Shape of Electrodes

The electrode shape plays an important role in determining the trapping capability when using DEP as well as the flow around it. For example, the use of sharp angles may lead to sharper electric field gradients and possible turbulence at high flow rates. Although the use of cylindrical electrodes is prominent in DEP [24,45,46], other shapes of electrodes or insulator structures have been studied. For example, Saucedo-Espinoza et al. explored insulator structures with diamond and circular cross-sections and width of ~80 μm. The use of a square shape was concluded to provide the highest trapping capacity due to the presence of large volumes with high electric field square gradient. However, the diamond and circle shapes were proven beneficial for cell focusing [47]. In the case of streaming DEP, Cummings et al. studied the use of diamond and circular cross-sections [48,49] In the case of diamond shaped electrodes, particle concentration was seen to deplete as the particles moved along the channel showing high rate of particle capture, whereas in case of posts with circular cross-sections, the particles became more focused as they moved through the channel but showed less capture than the diamonds.

The results obtained when using electrodes of different cross-sections but with their height equal to 100% of the channel height are shown in Figure 4a with a flow rate of 10 µL/min and cell concentration of 2 × 10^5^ cells/mL. The case of diamond cross-section yields a high amount of SC23 cells being trapped in the array due to the sharp corners of the electrodes and the high field gradients that are generated. Only 8.51% of the SC23 population input in the model could be retrieved and hence the predominance of blue in the figure. About 61.54% of the SC27 cells could be retrieved at the array exit. Although the retrieval throughput of SC27 is high, the purity is only around 90% in most regions, with 95% in a few regions. In the case of circle cross-section, the retrieval of SC27 is about 62.09% whereas SC23 streams have a throughput of 2.40%, showing less trapping of SC23 cells compared to diamonds. For circles, well defined regions with SC27 purity greater than 95% can be seen in the figure. The use of the lens cross-section yields a throughput of 69.43% for SC27 cells, comparable to that of circles, but features the least percentage of SC23 retrieval among the three, only 0.3% SC23, thereby indicating high trapping and the predominant white regions aligned with the electrodes. The purity obtained in the case of lens is higher than that obtained when using the other two cross-sections; close to 100% pure SC27 streams can be seen throughout the width of the channel.

### 4.2. Height of Electrodes

The height of the electrode with respect to the height of the channel was varied and studied to see the effect it has on the capture and effective focusing of the SC23 and SC27 streams. The obtained results are shown in Figure 4b when the electrodes are 10%, 50% and 100% of the channel height (100 µm here). These results were obtained when using circular electrodes, a flow rate of 200 µL/min and concentration of 2 × 10^5^ cells/mL. When the electrode height is 10% of the channel height, the throughput for SC23 is about 11.31%, whereas the throughput for SC27 is about 55.86%, indicating poor trapping for both cell types which is beneficial for continuous sorting. However, the average purity obtained at this electrode height for SC23 streams is around 20% (max. 25%), while the average purity for SC27 streams is around 70% (max. 75%). The electrodes with a height 10% of the channel are not a good choice for separation of the two species. For the electrodes with a height 50% of that of the channel, there is improvement in the trapping of the SC23 streams and the purity of SC27 streams. Around 3.37% of SC23 streams and 64.35% of SC27 streams can be retrieved. These electrodes show high trapping of SC23 streams, but the focusing of SC27 streams by negative DEP is limited. The case when the electrodes are as tall as the channel shows high improvement over the previous cases. Though the throughput of SC23 decreased to only 2.40% in this case, 68.09% SC27 streams were retrieved. The retrieval of highly focused SC23 and SC27 streams with purity higher than 95% is possible in some regions of the channel as seen in the graph. These results reinforce experimental results previously reported where the use of 3D carbon electrodes yielded better trapping efficiency [40].

### 4.3. Flow Rate

An important parameter to study when aiming at increasing device throughput is flow rate. The upper limit on the flow rate value is given by the necessity to prevent cell damage by shear stress. Hence, a maximum flow rate of 20 µL/min is used in this work. We report on the results obtained when increasing the flow rate from 5 to 20 µL/min at a concentration of 2 × 10^5^ cells/mL. Simulations using flow rate of 5 µL/min illustrate how all SC23 cells get captured due to dominance of dielectric force over the drag force. As the flow rate increases to 10 µL/min, the drag force starts to dominate the dielectric force and hence the number of trapped SC23 decreases, and around 2.40% of them can be retrieved. Up to 68.09% of SC27 streams are retrieved. Though the retrieval of SC27 streams is high, the purity in this case is affected as some SC23 streams are seen in the same region as the SC27. At 20 µL/min, the maximum purity in SC27 streams that can be expected is around 85% while the throughput is around 81.04%. The throughput of SC23 is 14.48% indicating the decrease in capture of SC23 as the flow rate increases from 10 µL/min to 20 µL/min. The average purity of SC27 streams is higher (85%–90%) in the regions closer to the electrodes than in the regions in between the electrodes where it is around 70%. This is because SC27 flowing near the electrodes are effectively diverted by the dielectric forces, whereas the SC23 streams in this region are attracted to the electrodes. These phenomena decrease as the cells flow deeper into the region between the electrodes, where the SC23 and SC27 are less affected by dielectric force, and more by the drag force. Being similar in size, there is no significant discrimination between them. At the flow rate of 20 µL/min, 100% pure SC23 can be obtained in regions immediately next to the electrodes. The dielectric force is very high in these regions and SC23 streams are directed towards the electrodes. However, given the high drag force at high flow rates, the SC23 cells no longer get captured but instead are focused and eluted collinearly to the electrodes. The SC23 cells that are not affected by the DEP force attracting them to the electrodes decrease the purity of the SC27 cell stream flowing in between electrodes.

### 4.4. Cell Concentration

The results obtained when simulating different cell concentrations at the flow rate of 10 µL/min when using 100 μm-high circular electrodes are shown in Figure 4d. The concentration increased from 10^2^ to 10^6^ cells/mL. The case with 10^2^ cells/mL corresponds to one cell flowing through the modeled channel at a given time. Similarly, 10^4^ cells correspond to 100 cells. For the case of 10^2^–10^3^ cells/mL, random coordinates were generated to determine the starting position of the cells. For the case of 10^3^ cells/mL, ten different coordinates were generated to obtain a more realistic model. Three repetitions were done for cases 10^4^ and 10^5^ cells/mL, while only one trial was implemented for the case of 10^6^ cells. The results shown in Figure 4d are representative of each case. In all cases, high purity SC27 streams are obtained from regions in between the electrodes, while mostly all the SC23s in the channel are trapped when concentration is less than 10^5^ cells/mL. At the concentration of 10^6^ cells of each type, the concentration is so high that not all SC23s are trapped anymore because the array starts to be saturated and the cells are eluted in characteristic streams linearly with the electrodes.

## 5. Discussion

The width of the elution stream, *T*, and the separating gap between the streams, *G*, are important design parameters in streaming DEP to enable continuous separation as shown in Figure 1. Depending on their values, one can design geometries with an optimized opening *W* for continuous extraction of the targeted cells from the channel. Photolithography is a technique that is amenable for the fabrication of such geometries [50]. If using photolithography, the minimal width of these retrieval geometries is likely to be in the tens of micrometers, with separation between each other in the same range. The width of these retrieval geometries, *W*, would ideally be less than the width of the cell stream, *T*, since this will allow for a buffer zone to mitigate contamination with other cells at the edges of the retrieval geometries. Although the downside to this will be the loss of targeted cells and a decrease in throughput, this may be less significant if the yield of cell expansion and differentiation during cell manufacturing is high.

**Figure 5:**
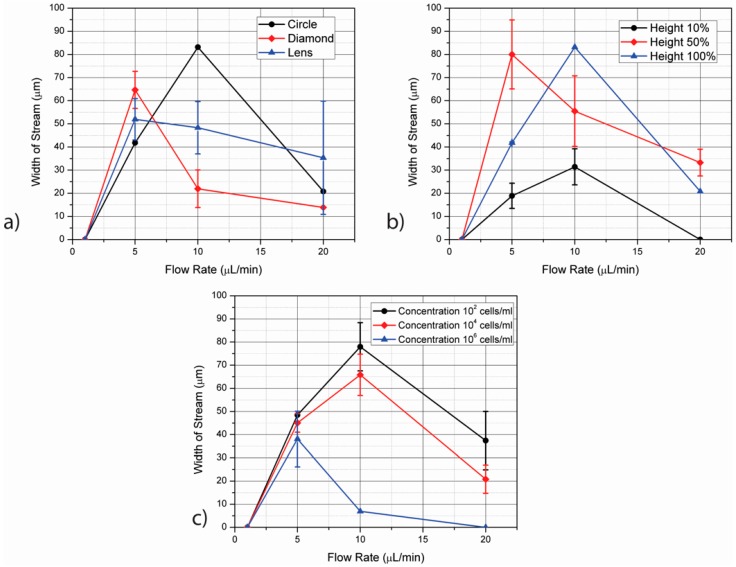
(**a**) The width of stream or zone obtained in the case of each electrode such that >98% pure streams of SC27 can be retrieved in the zone. The width of the zone initially increases with the increase in flow rate until it reaches a peak value before it starts decreasing. The circle shape features the widest zone for the flow rate of 10 µL/min followed by the lens and the diamond shape. However, the width of the zone decreases rapidly as the flow rate increases; (**b**) The width of the streams for obtaining >98% pure SC27 streams at different heights of the cylindrical electrodes as the flow rate varies. The stream width for the case of heights 50% and 100% of the channel are comparable as seen in this figure and are much higher than the case of height 10% of the channel. This reiterates the effect of height of the electrode for focusing. In this case, the stream width decreases with increase in flow rate; (**c**) The study of concentration of streams was conducted for stream purities of 98% and above with the increase in flow rates. The zone width increases with flow rate and shows a peak at the flow rate of 10 µL/min, after which it decreases. The zone width increases as the concentration of the cells decrease, mostly owing to less contamination of SC27 cells by SC23 cells that are retrieved through the zones.

The change of stream width according to the parameters studied here is presented in Figure 5. All points shown in this figure correspond to stream widths with purity >98% as calculated using Equation (12). Since the diameter of the SC27 cells is ~15 µm, streams narrower than this value are not considered. The effect of electrode cross-section is shown in Figure 5a. Similar to the results obtained when varying electrode height and cell concentration (Figure 5b,c, respectively), a peak on the stream width can be observed when varying the electrode cross-section. This peak occurs at 10 µL/min in the case of circular electrodes and 5 µL/min for diamonds and lens. Before these peak values, the increase on stream width is due to the combined effect of high trapping of SC23 cells on the electrodes and sharp focusing of SC27 streams by negative DEP. With the increase in flow rate beyond the corresponding peak values, not all SC23s are remain trapped and they start being eluted. The purity and width of the SC27 streams are thus reduced. At their peak values, the circular electrodes yield the widest retrieval zones at 83.16 ± 6.93 µm-width. Lens and diamonds yield 51.97 ± 8.94 µm and 64.68 ± 8.00 µm respectively at their peak values at 5 µL/min flow rate. The lens shape can be described as a combination between circle and diamond shape. Hence, it has regions with high gradients along the curved edges, like circles, which facilitate cell movement along the electrode surface. It also features sharp points like diamonds which are useful for positive DEP of SC23 due to the high electric field gradient generated at such points. In contrast to the case of diamonds where the stream width decreases rapidly after 5 µL/min of flow rate, the decrease in the case of lens is gradual. This is due to the focusing of SC23 towards the electrodes decreasing gradually when compared to diamonds.

A peak on the stream width can be observed when varying the other parameters of interest as well. In the case of electrode height, the use of electrodes as tall as the channel (100% height) leads to a maximum stream width of 83.16 ± 6.93 µm at 10 μL/min. The use of shorter electrodes does not yield stream widths as wide. In the case of electrodes spanning half the channel height, the peak value of stream width is seen at 5 µL/min but the value drops at higher flow rates since the drag force starts dominating the DEP force. The SC23 streams are not attracted to the electrodes as effectively and start flowing from the retrieval regions for SC27. The use of short electrodes only addresses the particles flowing close to the channel floor and hence their separation performance is poor. The region with active electric field is present only at the 10% of the channel height and hence the stream widths obtained in this case are smaller than the electrodes covering 50% and 100% of the channel height.

Using cell concentration of 10^2^ cells/mL results on the widest stream width (77.96 ± 10.39 µm) followed by 10^4^ cells/mL (65.83 ± 8.94 µm) and 10^6^ cells/mL (6.93 µm). A peak on the stream width is again observed at 10 μL /min for the concentrations of 10^2^ and 10^4^ cells/mL. An important observation here is with the cell concentration of 10^5^ cells/mL considered in the case of Figure 5a,b. In these cases, the cell concentration of 10^5^ cells/mL show a high stream width of around 83.16 µm. Thus the stream width is seen to decrease beyond this value of cell concentration. The use of flow rates of 20 μL/min leads to a significant decrease on the width of streams with >98% purity of SC27 cells. For a fixed stream width, a lower cell concentration ~10^2^ cells/mL with a high flow rate can give a device with high efficiency, but with a low throughput. Using samples with high concentrations ~10^6^ cells/mL at a low flow rate can give a high throughput but with a low efficiency. The parameters to be implemented can thus be optimized based on the requirement of throughput and efficiency depending on the application.

The behavior of stream width and composition depending on electrode geometry, flow rate and sample concentration has been studied here. Implementing a flow rate of 10 μL/min leads to SC27 cell streams featuring purity >98% and as wide as 80 μm when using cylindrical electrodes that are as tall as the channel and a sample concentration in the range 10^2^–10^5^ cells/mL. However, the throughput changes on the cell concentration, from 41.92% at 10^2^ cells/mL to 61.52% at 10^5^ cells/mL.

The number of cells currently required for neuron-based therapy is around 10^5^ per dose [51]. At a flow rate in the device of 10 μL/min, a sample concentration of 10^5^ cells/mL, a throughput of 62.09%, and assuming that up to 50% of the sample are cells of interest, the purification of SC27 cells using streaming DEP with the device modeled here will take around 6 h. At these conditions, an individual cell undergoes shear stress lower than 1.5 dynes/cm^2^. Future work will be on increasing the flow rate through the device while maintaining low levels of shear stress. For example, the channel cross-section will be significantly expanded to increase the flow rate thanks to the increase of cross-section only. This will likely require the fabrication of improved 3D electrodes and the optimization of their polarizing voltage depending on the electrode material.

Once the streams are formed, retrieval geometries will be implemented. The length of the retrieval geometry *L* in Figure 1 plays an important role to sustain the retrieved cells. A short geometry will cause quick accumulation and clogging of the retrieval geometry. Also, at high flow rates, the inflowing stream will cause transient flow in the collection geometry and will build back pressure in the zone. To avoid this, the retrieval geometry should be sufficiently long to allow continuation of the flow developed in the channel. The design of the entrance profile marked as *P* in the Figure 4 also affects the dynamics of the streaming geometry. For example, at a flow rate of 10 µL/min, a discrete separation between SC27 and SC23 streams is obtained and a pointed entrance profile will cut through the flow without affecting the cell streaming. The pointed entrance with the sloping edge facing the retrieval geometry can be used where the streams for pure SC27 cells are discretely defined and all the streams which are enclosed by the edges are collected. When the boundaries of the streams show a mixture of the two types of cells and it is desired to eliminate these cells, the sloping edge facing the outward side of the retrieval zone would enable their flow out of the geometry. With higher flow rates, the pointed profile will create obstructions in the flow and can create pressure drop or back flow as the stream enters the retrieval region, whereas the curved entrance profile will create a more gradual gradient of pressure as the stream transits from the channel to the retrieval geometry. Further investigations into the microchannel flow dynamics for the design of retrieval geometries are necessary and will be conducted in future work.

## 6. Conclusions

The results presented demonstrate the potential to continuously separate SC27 from SC23 cells using streaming dielectrophoresis (DEP). The impact of electrode geometry, flow rate and sample concentration on the generation of cell streams with purity >98% was studied. The use of circle and lens cross-section prove beneficial for obtaining high stream widths, but the circle cross-section offers better reproducibility. The use of electrodes as tall as the channel was concluded to be ideal due to the wide streams obtained at the flow rate of 10 µL/min. The stream widths show a peak with the increase in flow rate initially as the effect of DEP and drag forces aid the stream formation, but the stream width decreases further as the flow rate increases. Increasing the cell concentration above 10^5^ cells/mL was seen to generate narrower stream widths. The throughput obtained in the case of electrodes with 100% height and circular cross-section is 62.09% and varies with electrode shape and flow rate in the channel. The use of cylindrical electrodes as tall as the channel leads to the generation of streams with width up to 80 µm which facilitates the fabrication of retrieval geometries using photolithography. The time required to generate a therapeutic dose using the parameters optimized here can be as long as 6 h. This is deemed too long for a practical application and further work is required to shorten this time to less than 30 min. However, these initial results allow for identifying the effect of different parameters on the equilibrium between the DEP and drag forces required for streaming DEP. Streaming DEP can enable continuous separation of cells in a microfluidic chip. Further optimization of parameters like electrode shape, dimensions and flow rate are needed to decrease the processing times of relevant sample volumes for cell manufacturing.

## Figures and Tables

**Table 1:** The average percentage throughput in each of the cases in the simulation for SC23 and SC27 streams calculated using Equation (13). The throughput represents the percentage of total incoming streams of each type that are present in the streams after the electrode array. Since most of the SC23 streams show positive dielectrophoresis (DEP) and get captured at the electrodes, the throughput for this case is generally low. SC27 cells show negative DEP, no significant trapping and thus a high throughput is obtained.

Parameter Studied	Value	Throughput for SC23	Throughput for SC27
Shape of electrode	Lens	0.3%	69.43%
	Circle	2.40%	62.09%
	Diamond	8.51%	61.54%
Height of electrode as percentage of channel height	10%	11.31%	55.86%
	50%	3.37%	64.35%
	100%	2.40%	62.09%
Flow Rate	1 µL/min	0%	0%
	5 µL/min	0%	33.33%
	10 µL/min	2.40%	68.09%
	20 µL/min	14.48%	81.40%
Cell Concentration	10^2^ cells/mL	10.61%	41.92%
	10^4^ cells/mL	2.88%	68.18%
	10^6^ cells/mL	2.36%	62.52%

## Supplementary Materials

The following are available online at www.mdpi.com/2072-666X/7/12/217/s1. Table S1: Parameters defined for the simulation. Table S2: Equations defined for the simulation.
