# Supplementary material for: Numerical Model of Streaming DEP for Stem Cell Sorting
Source: Micromachines (Basel). 2016 Nov 30;7(12):217. doi: 10.3390/mi7120217 (PMC6190341; doi:10.3390/mi7120217)
Supplement: Supplementary file 1 [file micromachines-07-00217-s001.pdf]

Supplementary Materials: Numerical Model of Streaming DEP for Stem Cell Sorting

Rucha Natu and Rodrigo Martinez-Duarte

Table S1. Parameters defined for the simulation.

Name	Value	Description
Flow velocity	u	Flow rate in channel
R	From Figure 2a in the manuscript	Radius of Stem Cell
σ_m	0.51500 S/m	Conductivity of Media
CM	From Figure 2a in the manuscript	Clausius Mossotti Factor
μ	8.90×10^{-4} Pa·s	Dynamic viscosity of water as media at 25 °C
ε_m	80.2	Relative Permittivity of water as media

Table S2. Equations defined for the simulation. "ec" represents the equations used in Electric Currents interface in COMSOL.

Name	Expression	Unit	Description
GradEx	$d(\mathrm{ec}.E_y^2 + \mathrm{ec}.E_x^2 + \mathrm{ec}.E_z^2, x)$	$\mathrm{m{\cdot}kg^2/(s^6{\cdot}A^2)}$	Gradient in x direction
GradEy	$d(\mathrm{ec}.E_y^2 + \mathrm{ec}.E_x^2 + \mathrm{ec}.E_z^2, y)$	$\mathrm{m{\cdot}kg^2/(s^6{\cdot}A^2)}$	Gradient in y direction
GradEz	$d(\mathrm{ec}.E_y^2 + \mathrm{ec}.E_x^2 + \mathrm{ec}.E_z^2, z)$	$\mathrm{m{\cdot}kg^2/(s^6{\cdot}A^2)}$	Gradient in z direction
GradTotal	$\sqrt{\sum((d(\mathrm{ec}.E_y^2 + \mathrm{ec}.E_x^2 + \mathrm{ec}.E_z^2, x))^2}$	$\mathrm{m{\cdot}kg^2/(s^6{\cdot}A^2)}$	Total Gradient
FDragx	$6\pi R\mu \times u$	$\mathrm{m{\cdot}kg/s^2}$	Drag force in x direction (similar equations in y and z direction)
FDEPx	$2\pi R^3 \times \varepsilon_m \times$ CM × GradEx	$\mathrm{m{\cdot}kg/s^2}$	DEP Force in x direction (similar equations in y and z direction)
